# Visceral Leishmaniasis during Italian Renaissance, 1522–1562

**DOI:** 10.3201/eid1801.102001

**Published:** 2012-01

**Authors:** Andreas G. Nerlich, Raffaella Bianucci, Anna Trisciuoglio, Gabriele Schönian, Markus Ball, Valentina Giuffra, Beatrice Bachmeier, Carsten M. Pusch, Ezio Ferroglio, Gino Fornaciari

**Affiliations:** Hospital München-Bogenhausen, Munich, Germany (A.G. Nerlich);; University of Turin, Turin, Italy (R. Bianucci, A. Trisciuoglio, E. Ferroglio);; Charité Universitätsmedizin, Berlin, Germany (G. Schönian);; University of Tübingen, Tübingen, Germany (M. Ball, C.M. Pusch);; University of Pisa, Pisa, Italy (R. Bianucci, V. Giuffra, G. Fornaciari);; University of Munich, Munich (B. Bachmeier)

**Keywords:** leishmaniasis, visceral leishmaniasis, kala-azar, Leishmania, L. infantum, kinetoplast, mitochondrial DNA, Italian Renaissance, Medici, Eleonora from Toledo, Italy, parasites, protozoa

**To the Editor:** Leishmaniasis, an infectious disease caused by parasites of the genus *Leishmania*, is transmitted to humans through the bite of a female sandfly. The 3 forms of leishmaniasis are visceral (VL) and cutaneous (CL), which are typical of the Old World, and mucocutaneous leishmaniasis, which occurs primarily in Central and South America. VL (also called kala-azar) is caused by species of the *L. donovani* complex (including *L. infantum*), and CL is mainly caused by *L. major* or *L. tropica* (*1*). In Italy, VL and CL are caused by *L. infantum*. The origin and spread of leishmaniasis are a matter of debate. Widespread in antiquity, visceral leishmaniasis has been identified only in mummies from ancient Egypt and upper Nubia (*2*). Similarly, only 4 cases of mucocutaneous leishmaniasis have been identified in skulls from northern Chile (*3*).

We describe the identification of *L. infantum* infection in Eleonora from Toledo (1522–1562), wife of Cosimo I de’ Medici and member of one of the major political Italian families during the Renaissance. The positive identification of *Leishmania* infection was achieved in bone samples by 2 independent approaches. First, a molecular ancient DNA (aDNA) analysis identified a specific 123-bp fragment of a conserved region of the minicircle molecule of the parasite´s kinetoplastid mitochondrial DNA (*4**,**5*) which on direct sequencing showed a *Leishmania*-specific sequence compatible with *L. infantum* (Figure; Figure A1). This PCR result was independently replicated in 2 laboratories and additionally supported by the second approach, a protein assay showing a concomitant positive reaction by detecting IgG against *L. infantum* by Western blot sodium dodecyl sulfate–polyacrylamide gel electrophoresis.

Direct sequencing of the *Leishmania* aDNA identified a strain with high homology to *L. infantum*. Accordingly, we obtained a 98% concordance rate between our sequence and that of *L. infantum* (expect rate 6e-47, identity rate 113/118) (Figure A1). The rates for other *Leishmania* species indicated that concordance for those species was less probable.

For the protein assay, fractionated proteins from a lysate of late-log-phase promastigotes of *L. infantum* ZMON-1 (World Health Organization code MHOM/TN/1980/IPT-1) were electroblotted onto nitrocellulose membrane, and antibody detection was conducted on a Bio-Rad (Hercules, CA, USA) Multiscreen apparatus (*6*). Antibodies against *L. infantum* selectively reacted in a supernatant of protein extract from Eleonora, thereby confirming the immunologic identification of the protozoal infection. The response of IgG against *L. infantum* whole-parasite antigens revealed specific recognition of 8 polypeptides ranging from 14–16 kD to 184 kD. This pattern of bands is consistent with a symptomatic form of VL as shown by the 14 to 16–kD bands.

Although it was initially proposed that the antigenicity of ancient proteins may be altered by diagenesis, further investigations have shown that ancient immunoglobulins can persist across geologic times (*7*). Potential pitfalls in protein-based detection of ancient pathogens have been addressed by incorporating proper controls during the analysis. False-positive data, which can result either from contamination of ancient material by modern materials or from lack of microbe specificity of the test (*7*), have been ruled out by the parallel testing of several blanks (buffer without ancient material) and by testing, in parallel, samples of ancient bone tissue harvested from persons who died of known diseases other than leishmaniasis (e.g., plague). All negative controls used in aDNA and protein research and all blanks yielded negative results. To avoid contamination, we used no positive controls.

The disease history of Eleonora from Toledo is as follows. Her clinical history was dominated by a large number of pregnancies. When 18–32 years of age, she gave birth to as many as 11 infants. On the basis of additional clinical reports of court doctors, it was assumed that pulmonary tuberculosis developed when she was 29 years of age (*8*). In the last years of her life, Eleonora from Toledo had various severe ailments. Irregular bouts of fever, wasting and constant vomiting, stomach pain, weight loss, anemia, and hemorrhage were recorded. Autopsy revealed that her most damaged organs were the lungs and that the lung lesions were consistent with a chronic pulmonary infection. Hepatomagaly and splenomegaly were also recorded (*9*). Although these signs and symptoms could have come from the tuberculosis infection, they are also consistent with those in patients with symptomatic VL, i.e., progressive fever, weight loss, splenomegaly, hepatomegaly, hypergammaglobulinemia, and pancytopenia. Complications include immunosuppression, secondary bacterial infections, hemorrhage, and anemia (*10*). All these observations lend support to the notion that Eleonora from Toledo was not immunocompetent. In addition to a supposed tuberculosis co-infection, VL infection may have been a key event leading to her death at age 40.

Our molecular and serologic identification of *Leishmania* infection in a historically prominent person from southern Europe has major relevance. This information might be useful for monitoring the infection and its pathogen throughout history and might provide data on the host–pathogen interaction over different periods.

**Figure:**
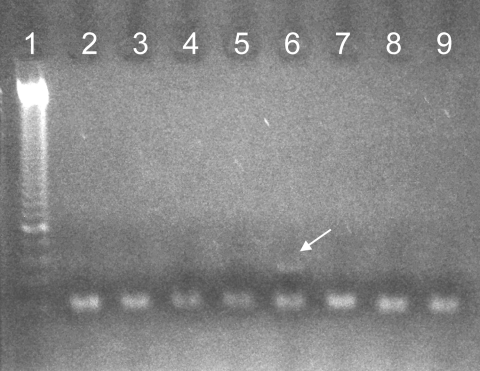
PCR amplification of a 123-bp fragment of kinetoplastid mitochondrial DNA of *Leishmania* spp. from Eleonora from Toledo (lane 6, arrow). Lane 1, molecular mass standard; lanes 2–5, ancient controls; lanes 7–9, blank controls.

## Figures and Tables

**Figure A1:**
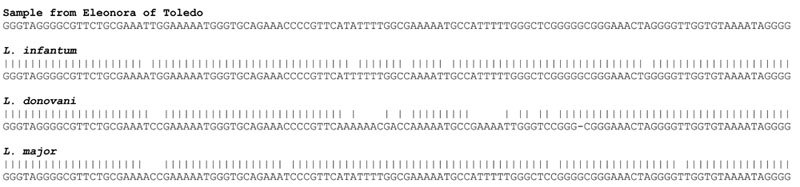
Sequence alignment showing high but not complete homology to *Leishmania infantum.* Subj, the sequence from Eleonora of Toledo.
